# Romanian GPs Involvement in Caring for the Mental Health Problems of the Elderly Population: A Cross-Sectional Study

**DOI:** 10.3389/fneur.2021.641217

**Published:** 2021-06-24

**Authors:** Raluca Sfetcu, Daciana Toma, Catalina Tudose, Cristian Vladescu

**Affiliations:** ^1^Psychology Department, “Spiru Haret” University, Bucharest, Romania; ^2^The Center for Health Services Assessment and Research, National School of Public Health, Management and Professional Development, Bucharest, Romania; ^3^National Society for Family Medicine, Bucharest, Romania; ^4^Psychiatry Department, University of Medicine and Pharmacy “Carol Davila”, Bucharest, Romania; ^5^Public Health Department, Faculty of Medicine, “Titu Maiorescu” University, Bucharest, Romania

**Keywords:** mental health, primary care, elderly, roles, barriers

## Abstract

The mental health of the elderly is a matter of increased concern in the context of an aging population since currently only a small fraction of this population is receiving adequate care. The provision of treatment in primary care by the General Practitioners (GPs) has been proposed for over a decade as a potential solution, as services offered by GPs are more accessible, less susceptible to stigma, and have a more comprehensive view of the other health care problems that the elderly might suffer from. In this study, we explored the perception of Romanian GPs regarding their practice and roles in caring for the mental health of the elderly as well as the willingness to increase their future involvement in the management of dementia and other mental health problems. Data was collected via an online questionnaire structured on four dimensions: (1) GPs' sociodemographic profile and practice characteristics, (2) GPs assessment of the services available for elderly with mental health problems, (3) GPs current involvement in mental health care for different categories of problems, and (4) factors that might influence the future involvement of GPs in providing care for elderly with mental health problems. The survey was sent via the member mailing lists of the National Society for Family Medicine. Results show that GPs are currently limited by prescribing possibilities, available resources and knowledge in the area, but they are willing to expand their role in the areas of early recognition and prevention of mental health problems as well as providing disease management and collaborative care. An improved communication with mental health care professionals, a better access to resources and having more financial incentives are the three most important categories for GPs to increase their involvement. In conclusion, increasing the access to personal and professional resources and setting up functional communication channels with specialized mental health care could motivate GPs to provide timely mental health support to elderly patients.

## Introduction

The mental health of elderly is of increasing concern in an aging population context, with 20% of adults aged 55 and over suffering from a mental disorder of which most commonly reported are anxiety disorders, severe cognitive impairment, and mood disorders. Nevertheless, the majority of this population does not receive the services they need, with 70% of older adults not seeking help from a mental health professional for their mental health problems (1, 2). This represents an especially important issue in light of the accumulated knowledge that all mental health disorders adversely affect physical health. Studies show that untreated depression in the elderly who also suffer from heart disease can negatively affect the outcome of the somatic condition (3). However, currently depression is still widely under detected in older people, with only one in six elderly people with depression actually discussing their symptoms with the GP and less than half of them receiving adequate treatment (4). The situation was found to be similar for elderly suffering from anxiety disorders (5, 6) and in Low and Middle Income Countries (LMIC) the treatment gap is estimated to be even higher (7). Despite the obvious impact that untreated mental health problems might have (8), the gaps in the provision of services for elderly still exist due to factors such as: the stigma surrounding mental illness and mental health treatment; denial of problems; access barriers; fragmented and inadequate funding for mental health services; lack of collaboration and coordination among primary care, mental health, and aging services providers; gaps in services; and the lack of adequate professional and paraprofessional staff trained in the provision of geriatric mental health services (9).

Since ~70% of the population seeks health care in primary care settings (10) and General Practitioners (GPs) are frequently the gatekeepers to specialized mental health services (11), they could play an important role in addressing this detection and treatment gap.

Recent research conducted in Indonesia and other LMICs show that GPs supported by nurses in primary care clinics could effectively manage mild to moderate mental health issues commonly found among primary care patients and that they provide non-stigmatizing mental health care within community context, helping to reduce the mental health Treatment Gap (12, 13). While the idea of increasing the involvement of GPs in the treatment of the elderly with mental health problems is at least a decade old, it has been suggested that the feasibility of such an approach must be tested in each country (12).

In Romania such an approach has not been yet tested, although previous efforts to increase the knowledge of GPs in the area of mental health care have been made (14). GPs are currently reimbursed mainly on capitation, with very few fee-for services, none of them for mental health care (15) and they have limited capacity in the prescription of drugs for mental health disorders. They are generally being asked to continue the prescriptions initiated by psychiatrists for a limited period of time (usually up to 3 months) after which a new prescription from the psychiatrist is needed (16). The current legislation also limits the initiation and continuation of the treatment for dementia to specialists such as psychiatrists, neurologists, and geriatricians; these are the main providers of care either in hospitals or outpatient services as no specialized services are currently available for the elderly with mental health problems (17). The official role of the GPs for this type of diagnosis is to identify patients and refer them to the specialists. However, access to specialized services can be difficult in many regions of the country and—when available—these might not meet the actual needs which can result in multiple consultations (18).

In this context, we aimed to explore the views of Romanian GPs on their current role, on factors that deter them from playing a more active role as well as on the specific areas where they would be willing to intensify their involvement in the mental health care of the elderly. Through this study we aim to understand how GPs perceive the services currently provided to elderly with dementia and other mental health problems, their current place within the mental health care system as well as enabling and hindering factors for a further expansion of their role. We hope our results will be used to inform future health care policies in this area.

## Methods

### Study Design

This is a cross-sectional survey-based, observational study on the perceived current and future role of GPs in the area of the mental health care of the elderly.

### Participants

The selection of participants was opportunistic. The questionnaire was made available in a digital format and a link was posted by one of the authors on the internal communication channels of the national GPs professional society [i.e., National Society of Family Doctors (NSFD)]. The announcement was available online for 2 weeks in May 2018 and resulted in 65 completed questionnaires. Around 4,000 GPs from all over the country are subscribed to the NSFD communication group where the announcement was posted; however due to individual message viewing preferences pre-set by GPs, we cannot estimate the actual number of members who have actually seen our announcement and we cannot give an estimate of the actual response rate.

### Instruments

The questionnaire was constructed by the research team, based on a selective review of the literature (19–27), as further indicated below. The structure of the questionnaire reflects our goal to cover different aspects that might influence the current and future role of GPs in providing mental health service to elderly population while keeping the response time around 15 min (no financial incentive was provided to respondents for their participation). We have tested and refined the questionnaire by asking four key experts (three GPs and one psychiatrist) to fill in the form and make suggestions for improvement, wherever necessary.

The questionnaire covers four dimensions: (1) GPs' sociodemographic profile and practice characteristics, (2) GPs assessment of the services available for elderly with mental health problems, (3) GPs current involvement in mental health care for different categories of problems, and (4) factors that might influence the future involvement of GPs in providing care for elderly with mental health problems. It includes categorical or continuous items, with 4- or 5 point Likert-scale questions. Elderly population was defined as people aged 65 years old and over.

(1) *GPs' sociodemographic profile and practice characteristics*; this dimension included five questions.

As demographic variables, age, and sex were surveyed, as well as the place of the GP office. For the location of the practice, the answer options were “big city,” “suburbs,” “small town,” and “rural area” (these categories were dichotomized into urban (big cities, suburbs, and small town) and rural (mixed urban-rural and rural) areas). In terms of the characteristics of the practice we have asked GPs about their practice setting (whether working alone, with other GPs, with other medical specialties or with GPs and other medical specialties in a shared space) as well as how they estimate their workload to be (working below capacity, at capacity or above capacity). These items have been taken over and adapted from the QUALICOPC study (19, 20).

(2) *GPs assessment of the services available for elderly with mental health problems*; two questions were used to assess how GPs perceive the current provision of services for the elderly.

The first question asked the GPs to rate on a four point Likert scale the availability, diversity, continuity and the global quality of the mental health care services currently available to elderly (21, 22) and the second asked them to rate on five point Likert scale the degree to which different categories of services currently available to elderly with mental health problems respond to their needs. The services included were: General Practitioners, Outpatient psychiatric services, Outpatient neurologic services, Outpatient geriatric services Community Mental Health Center, Day centers, Psychiatric hospitals, Psychiatric departments in general hospitals, Geriatric departments in general hospitals, Neurologic departments in general hospitals, Residential centers, Nursing homes, Memory centers, Home care services, and Palliative care services.

The second question was developed by the authors to reflect the structure of the current provision of services for the elderly in Romania.

(3) *GPs as first contact for psycho-social problems and their current involvement in the initiation and treatment of mental health care for different categories of patients*; this dimension included two questions.

The first referred to the extent to which seven different categories of patients contact the GP as first health care provider (e.g., Man aged 68 with neurocognitive problems (e.g., dementia), Woman aged 70 with anxiety and depression problems, etc.) with the answer categories “almost always,” “usually,” “occasionally,” or “seldom/never.”

The second inquired about the involvement of the GP in the initiation and treatment of anxiety disorders, depression, personality disorders, schizophrenia, Parkinson disease, Alzheimer disease, and other neurodegenerative disorders.

(4) *Enabling and hindering factors for GPs future involvement in providing care for elderly with mental health problems*; this dimension included three subsections.

One addressed the barriers that prevent GPs for assuming a more active role in caring for the mental health problems of the elderly and included the following six categories: access to specialized services (4 items), communication with mental health care providers (6 items), personal and professional resources (3 items), patient related factors (2 items), financial related factors (4 items), and competencies (11 items). The list of factors has been created by the authors, based on previous research conducted in this area (21–23, 26, 27).

The second subsection asked about GPs willingness to get involved in different areas of mental health care for the elderly population such as: early recognition and prevention (1 item), self-management and e-health interventions (2 items), diagnosis and treatment (7 items), disease management and collaborative care (2 items), and relapse prevention, rehabilitation, and participation (2 items). This section was developed based on the stepped care model (24).

The last question prompted GPs to self-assess their abilities to provide care for anxiety disorders, depression, personality disorders, schizophrenia, Parkinson disease, Alzheimer disease, and other neurodegenerative disorders both in the current context as well as in an ideal context, where the current barriers would be removed.

### Data Analysis

Data were analyzed using descriptive statistics for all included variables (frequencies, percentages, means, and standard deviations). We have also performed a correlation analysis of the different areas of care that GPs would be willing to get involved in with the categories of factors indicated as barriers. To perform the analysis, we have used the Statistical Package for the Social Sciences (SPSS) for Mac, version 20.0.

### Ethical Issues

The ethical approval was obtained from the “Spiru Haret University” ethical review board and the protocol was determined to be exempt. No harm was anticipated to the survey respondents. The consent letter was placed directly within the survey, before the actual survey questions. At the end of the consent letter, participants had to provide their consent to participate in the study.

## Results

### GPs' Sociodemographic Profile and Practice Characteristics

Participants to this study were 65 General Practitioners (GPs), having an average age of 51.6 years (SD = 10.6) and living in 22 counties in Romania (32 from Bucharest, three each from Bihor, Dolj and Prahova, two each from Calarasi, Ilfov, Satu Mare, Teleorman, Tulcea and Valcea and one from Arad, Bacau, Botosani, Braila, Brasov, Cluj, Galati, Gorj, Hunedoara, Ialomita, Iasi, Vrancea). The majority (89.2%) were female practicing in a big city (64.6%) in an individual office or together with other GPs (77%). Half of the participants reported working above their capacity level, with an average size of the practice population of 2032 (SD = 728) patients on the list (see Table 1). The demographic structure of our sample is similar to ones included in other studies, a WHO report on the structure and provision of primary care in Romania reporting an average age of 49.5 years and 80% of respondents being female (28).

**Table 1:** General practitioner (GP) characteristics and aspects of their practice and interprofessional collaboration.

**Demographic data (*N* = 65)**	***N* (%)**
**Gender**	
Male	5 (7.7)
Female	58 (89.2)
**Where is the practice located**	
Big (inner) city	42 (64.6)
Suburbs	1 (1.5)
(Small) town	7 (10.8)
Mixed urban-rural	3 (4.6)
Rural	12 (18.5)
**Practice setting**	
Working alone	30 (46.2)
With other GPs in a shared space	20 (30.8)
With other medical specialties in a shared space	13 (20.0)
With GPs and other medical specialties in a shared space	2 (3.1)
**Workload**	
I still have time	10 (15.4)
I work at my capacity level	22 (33.8)
I work above my capacity level	33 (50.8)

### GPs Assessment of the Services Available for Elderly With Mental Health Problems

When asked about the mental health services for elderly people with mental health problems, the vast majority of participants have indicated that these are poor or very poor in terms of diversity (89.2%), availability (86.2%) and continuity (83.1). For the global quality of the services, a slightly higher number of participants (i.e., 21.5%) are of the opinion that the services are of good or very good quality (see Table 2).

**Table 2:** GPs' perception of mental health care services for the elderly in terms of availability, diversity, continuity, and global quality.

**GPs' perception of mental health care services** **for the elderly in terms of *N* = 65**	**Poor and very poor** ***N* (%)**	**Good and very good** ***N* (%)**
Availability (Number)	56 (86.2)	9 (13.8)
Diversity	58 (89.2)	6 (9.2)
Continuity	54 (83.1)	11 (16.9)
Global quality	48 (75.4)	14 (21.5)

A more detailed image of how the performance of different services is perceived by the GPs, shows that outpatient psychiatric services meet the mental health needs of the elderly in a high or very high degree (50.8%). GPs are following closely (46.2%), being the second most responsive professional category followed by neurologic outpatient services (36.9%), psychiatric hospitals (27.7%) and community mental health centers (23.1%). The mental health needs of the elderly are addressed in a moderate way by inpatient psychiatric, neurologic and geriatric services and poorly by residential care, nursing homes, home care, memory centers, day centers or palliative care services (for details, see Table 3).

**Table 3:** GP's assessment of the degree to which services available to elderly with mental health problems respond to their needs.

**How well are the following services responding to the mental health care needs of the elderly (*N* = 65)**	**Not at all or to a low degree *N* (%)**	**Some degree *N* (%)**	**High or very high degree *N* (%)**
General practitioners	6 (9.2)	29 (44.6)	30 (46.2)
Outpatient psychiatric services	10 (15.4)	22 (33.8)	33 (50.8)
Outpatient neurologic services	14 (21.5)	27 (41.5)	24 (36.9)
Outpatient geriatric services	23 (35.4)	12 (18.5)	7 (10.8)
Community mental health center	25 (38.5)	22 (33.8)	15 (23.1)
Day centers	34 (52.3)	14 (21.5)	9 (13.8)
Psychiatric hospitals	26 (40.0)	19 (29.2)	18 (27.7)
Psychiatric departments in general hospitals	23 (35.4)	22 (33.8)	15 (23.1)
Geriatric departments in general hospitals	28 (43.1)	15 (23.1)	14 (21.5)
Neurologic departments in general hospitals	27 (41.5)	23 (35.4)	13 (20.0)
Residential centers	28 (43.1)	19 (29.2)	4 (7.7)
Nursing homes	32 (49.2)	17 (26.2)	10 (15.4)
Memory centers	22 (33.8)	16 (24.6)	10 (15.4)
Home care services	30 (46.2)	18 (27.7)	10 (15.4)
Palliative care services	27 (41.5)	20 (30.8)	8 (12.3)

### GPs Current Involvement in Mental Health Care

A high number of GPs (81.5–98.5%) report that patients with psychosocial problems, anxiety, depression, memory problems and other neurocognitive problems are frequently contacting them, as their first health care provider. Elderly female patients with anxiety and depression problems are the category for which the GPs are almost exclusively the first health care provider (i.e., 98.5%). The next most frequent categories are elderly males and females with memory problems and/or other neurocognitive problems (Table 4).

**Table 4:** Reported categories of patients who contact their GP as the first health care provider for different mental health care problems.

**First option (contact for *N* = 65)**	***Rarely***	***Frequently***
	***N* (%)**	***N* (%)**
Man aged 28 with a first convulsion	44 (67.7)	21 (32.3)
Anxious man aged 45	9 (13.8)	56 (86.2)
Woman aged 50 with psychosocial problems	12 (18.5)	53 (81.5)
Woman aged 70 with anxiety and depression problems	1 (1.5)	64 (98.5)
Man aged 52 with alcohol addiction problems	32 (49.2)	33 (50.8)
Woman aged 75 with moderate memory problems	4 (6.2)	61 (93.8)
Man aged 68 with neurocognitive problems (e.g., dementia)	7 (10.8)	58 (89.2)

These results are also confirmed by the high frequency of cases where GPs initiate or provide treatment for mental health problems, anxiety disorders being the most mentioned category (i.e., 73.8%) followed by depression (52.3%). The involvement of GPs in the treatment of patients with Alzheimer disease or other neurodegenerative problems is reported by only one in four GPs to be a frequently occurring practice. The involvement in treating serious mental illness is also reported only by a few GPs (10.8% for Schizophrenia and 18.5% for personality disorders; see Table 5).

**Table 5:** Involvement in initiation and treatment of different mental health problems.

**Current involvement for the following categories**	***Rarely***	***Frequently***
	***N* (%)**	***N* (%)**
Anxiety disorders	17 (26.2)	48 (73.8)
Depression	31 (47.7)	34 (52.3)
Personality disorders	53 (81.5)	12 (18.5)
Schizophrenia	58 (89.2)	7 (10.8)
Parkinson disease	51 (78.5)	14 (21.5)
Alzheimer disease	49 (75.4)	16 (24.6)
Other neurodegenerative disorders	49 (75.4)	16 (24.6)

### Enabling and Hindering Factors for GPs Involvement in Providing Care for Elderly With Mental Health Problems

The main barrier to getting involved in the treatment of elderly with mental health problems that was mentioned by the participants to our study is the communication with mental health care providers (psychiatrists, psychologists, members of community mental health care teams), followed by the limited access to specialized mental health care services (i.e., difficulty in accessing specialized resources, a scarcity of mental health care workers, inadequate support and communication among stakeholders or a lack of critical mental healthcare resources). The third category of factors perceived as playing an important role, is represented by financial related factors (i.e., inadequate level of remuneration, increased bureaucracy to get reimbursed, investments of education and lack of financial motivation). Mean scores for all categories measured are presented in Table 6. While all factors are perceived as being important, the lowest mean score is corresponding to the category of personal and professional resources. Nevertheless, when looking at individual factors, the limited possibility to prescribe medication is reported by over half of the participants to be in a very high degree a barrier to their involvement in treating the mental health problems of the elderly. Other individual factor for which GPs agreed that play a very high role were the uncertainty of the diagnosis (44.6%), a lack of support from mental healthcare teams (43.1%), the bureaucracy to get reimbursed (41.5%), the inadequate level of remuneration (40.0%) and the lack of explicitness in the roles of different healthcare professionals (e.g., GPs, psychiatrists, psychologists) in managing mental conditions. Scores for all individual factors are included in Appendix A).

**Table 6:** Categories of factors that represent barriers to getting involved in the management of mental health problems of the elderly population.

**Categories of factor that represent barriers to mental health care**	**Mean (*SD*)**
(F1) Access to specialized services	3.75 (.94)
(F2) Communication with mental health care providers	3.85 (.80)
(F3) Personal and professional resources	3.40 (1.05)
(F4) Patient related factors	3.50 (1.03)
(F5) Financial related factors	3.72 (1.04)
(F6) Competencies	3.56 (.80)

While GPs are reporting above average interest to get involved in all areas of mental health care for the elderly, they would be willing to invest more effort into early recognition and prevention of mental health problems as well as providing disease management and collaborative care (i.e., referring to other health care providers and continuing care initiated in secondary care). The least interesting area for GPs was the one focusing on providing support for self-management or prescribing e-health interventions (for details, see Table 7 and Appendix B).

**Table 7:** Willingness of GPs to get involved in different areas of mental health care of the elderly population.

**Areas of mental health care** **(elderly population)**	**Mean (*SD*)**
Early recognition and prevention^a^	4.30 (.91)
Self-management and e-health interventions	3.68 (1.07)
Diagnosis and treatment^b^	3.80 (.85)
Disease management and collaborative care^c^	4.03 (1.00)
Relapse prevention, rehabilitation and participation^d^	3.80 (1.05)

a *Correlates with F2 (0.05 level);*

b *Correlates with F3 (.05 level);*

c *Correlates with F2 (.01 level) and with F1, F3, and F5 (0.05 level);*

d *Correlates with F2 and F3 (0.05 level)*.

To identify which factor might have an influence on the willingness of GPs to get involved in different areas of care we have run a correlation analysis between the six categories of factors discussed above (see Table 6) and the five areas of mental health care of the elderly population. Our results show a positive correlation between the area of “early recognition and prevention” and the scores obtained for the “communication with mental health providers” indicating that a better communication with the mental health providers would improve the willingness of GPs to get involved in “early recognition and prevention” (significant at 0.05 level). Similarly, we have found significant corelation between the area of “diagnosis and treatment” and “the level of personal and professional resources” (0.05 level). For the area of “disease management and collaborative care” we have found correlations with “communication with mental health providers” (0.01 level) as well as with the “access to specialized services” and “the level of personal and professional resources” (0.05 level). Finally, the area of “relapse prevention, rehabilitation and participation” correlates with “communication with mental health providers” and “the level of personal and professional resources” (0.05 level).

A last set of questions we have asked the GPs was addressing their ability to manage the mental health problems of the elderly in context of the existing barriers as well as in an ideal context, where most of these barriers would be removed. As expected, the current ability was assessed to be the highest for anxiety and depression and the lowest for the severe mental illnesses (schizophrenia and personality disorders). For Parkinson and Alzheimer diseases the current ability was estimated as low, with just one in three GPs believing that they would have the skills needed to manage this category of problems (see Table 8). However, these are the same diseases for which GPs expect the highest increase in ability, in the scenario where more resources would be available to them (26.1 and 27.7%, respectively).

**Table 8:** Self-assessment of GPs abilities to provide care for different categories of mental health problems in the current context and in an ideal context.

**Categories**	**Current context**	**Ideal Context**	**Increase %**
	**YES [*N* (%)]**	**YES [*N* (%)]**	
Anxiety disorders	57 (87.7)	62 (95.4)	7.7
Depression	46 (70.8)	58 (89.2)	18.4
Personality disorders	6 (9.2)	20 (30.8)	21.6
Schizophrenia	5 (7.7)	15 (23.1)	15.4
Parkinson disease	20 (30.8)	37 (56.9)	26.1
Alzheimer disease	22 (33.8)	40 (61.5)	27.7
Other neurodegenerative disorders	14 (21.5)	30 (46.2)	24.7

## Discussion

The aim of this study was to shed light on how GPs perceive the services currently provided to elderly with mental health problems, their place within the care system as well as the enabling and hindering factors for a possible future expansion of their role. We have discovered that GPs have an accurate understanding of the existent gap in the provision of mental health care for the elderly in terms of the number, the diversity and the continuity of the available services. For each of these dimensions over 75% of the participants estimate that the current provision is poor or very poor. These percentages are very high by comparison with data from other countries, a similar study from Canada indicating a dissatisfaction with the overall quality of the mental health care system of only 45% (diversity: 46%, continuity: 54%, and availability: 65%) (21). GPs also believe that services that are best meeting the needs of the elderly are provided in psychiatric and neurologic outpatient settings, psychiatric hospitals and by GPs themselves. Taking into consideration the fact that these are the most widespread institutional and human resources in Romania (17), it is highly probable that the majority of mental health services provided to the elderly is indeed taking place in these four main types of services.

The majority of the GPs in our sample (81.5–98.5%) report being frequently the first health care provider for patients with psychosocial problems, anxiety, depression, memory problems, and other neurocognitive problems. However, we have found that not all of these GPs initiate or provide treatment for anxiety disorders (73.8%) or depression (52.3%). As GPs in Romania play a gatekeeping role (29), these results are to be expected and are consistent with a recent study who shows that GPs are the first point of care for 66% of adults with anxiety problems and for 84% of elderly with memory problems (30). The same study indicates that GPs are involved in the treatment of follow-up of the depression in the general population in 74% of cases. These differences might indicate that GPs are less confident or are less motivated to treat the depression of the elderly than treating depression in adults (31), or they might even view it as “justifiable depression” (32). However, this aspect should be adequately investigated in future research.

One of the categories of factors that might have an impact on the decision to get involved in the care of elderly with mental health problems is represented by the lack of communication with mental health care providers. These findings are reinforced by results of Butu et al. (30), where 55% of the Romanian GPs investigated have reported to have rarely or never asked advice from a psychiatrist or have met them face to face. Our results are also similar with those of studies conducted in other countries which highlight that, in general, GPs are willing to initiate and provide treatment for mental health problems if they would have better collaborative relationships with mental health specialists (33–35). For example, a study conducted in France found out that a minority of GPs had a satisfactory relationship with private psychiatrists (49.5%) and public psychiatrists (36). As many different models of collaboration and communication have been proposed in the literature, an in-depth analysis of what is the best form of direct communication with other professionals and what represents an optimized referral process would increase the understanding of this issue. As virtual communication methods are rapidly expanding and cost-effective interventions for improving the collaboration between GPs and other specialists could be easily developed, this is also a timely exploration.

Having access to specialized mental health resources and a more advantageous financing model for services provided by GPs can also incentivize GPs for providing treatment to elderly with mental health problems. These findings are not specific for Romania, as access to specialized resources, the inadequate methods of remuneration and lack of financial incentives for GPs to manage mental disorders are frequently mentioned in the literature as hindering factors (26, 31). The need for the development of evidence-based resources for older people with mental health problems within primary care was recognized as a highly needed intervention (32). However, due to the differences in how health care systems are organized, solutions must be developed locally and in close collaboration with all actors involved. The future exploration of these issues should include qualitative research with GPs, psychiatrists and representatives of financing bodies.

In terms of the skills GPs need in order to provide mental health services to an elderly population, the strong areas are represented by depression and anxiety disorders. For the rest of diagnostic categories, the percentages of GPs reporting a good level of skills is rather low. However, in an ideal context where current barriers would be surpassed, the GPs expect an increase of these skills of up to 25%, which represent an encouraging result. Previous studies also highlight the fact that GPs are well-positioned to address MHP in their older patients because of (a) their long-lasting bond with their older patients, (b) their holistic view of the patient, and (c) the easy low-threshold access they offer, but the lack of knowledge, skills, and confidence in their skills can substantially impair detection as well as treatment (33). In Romania, interventions for increasing the skills of GPs have been implemented in the last decades but these were project based and did not lead to systemic changes in the initial training of GPs, in the Continuing Medical Education (CME) programs or in the financial incentive structure. Connecting the training with financial incentives have proved to be successful strategies to increase mental health services provision, as demonstrated by the “Better Outcomes” program implemented in Australia between 2000 and 2007 and should be included in future interventions (37).

Despite the above discussed barriers, GPs are reporting an above average interest to get involved in all areas of mental health care for the elderly. However, they would be willing to invest more effort into early recognition and prevention of mental health problems as well as providing disease management and collaborative care. These results indicate that increasing the access to personal and professional resources and setting up functional communication channels with specialized mental health care could increase the willingness of GPs to provide timely mental health support to a larger number of elderly populations. One additional factor to be considered when planning for the involvement of GPs in the treatment of mental health problems of the elderly is their limited time availability, as mental health care can be time consuming and half of our participants have reported already working above their capacity level (22).

Taking into consideration the limited number of participants as well as the opportunistic method used for sampling them, the results of this study should be used with caution. The fact that we have not distributed the questionnaire directly but we have used the communication channels of the National Society for Family Medicine for their regular contact with members might have played a role in the decision of GPs whether to take part in the research or not, even before opening the email (were more information about the study was included). This might be a possible explanation for the low response rate. Also, as GPs had the possibility to self-register for this study it is also likely that GPs with a higher interest for the topic have answered the questionnaire. Despite the limitations and biases associated with this data collection method, it was the only available method of reaching a large number of GPs without violating the data protection rules applicable at the moment of data collection. Despite these limitations, our results can be used to inform future studies with representative samples, which are much needed by decision makers for development of future strategies.

## Conclusions

Our study has found that communication with other mental health care professionals, access to resources and financial incentives are the three most important factors which influence their involvement in providing mental health care for the elderly. While still limited by prescribing possibilities, available resources and knowledge in the area, GPs are willing to expand their role in the areas of early recognition and prevention of mental health problems as well as in providing disease management and collaborative care. Increasing the access to personal and professional resources and setting up functional communication channels with specialized mental health care could motivate GPs to provide timely mental health support to elderly patients. Strategies in the area of service development should also take into consideration the development of adequate financing mechanisms and financial incentives. Future research should focus on the aspects such as: comparative analyses between the willingness of GPs to initiate and provide treatment for mental health care problems to the elderly population by comparison with the general/adult population, understanding what optimal communication with other specialists and an optimized referral process should look like, and preferred method of providing access to specialized mental health resources (e.g., training, best practice/guidelines, and other types of tools).

## Data Availability Statement

The original contributions presented in the study are included in the article/Supplementary Material, further inquiries can be directed to the corresponding author/s.

## Ethics Statement

The studies involving human participants were reviewed and approved by Ethical Committee of the Spiru Haret University. The patients/participants provided their online consent to participate in this study.

## Author Contributions

RS, DT, CT, and CV: conceptualization and writing—review and editing. RS, DT, and CT: methodology. RS: writing—original draft preparation. All authors have read and agreed to the published version of the manuscript.

## Conflict of Interest

The authors declare that the research was conducted in the absence of any commercial or financial relationships that could be construed as a potential conflict of interest.
