# Supplementary material for: Romanian GPs Involvement in Caring for the Mental Health Problems of the Elderly Population: A Cross-Sectional Study
Source: Front Neurol. 2021 Jun 24;12:641217. doi: 10.3389/fneur.2021.641217 (PMC8263930; doi:10.3389/fneur.2021.641217)
Supplement: Supplementary file 1 [file Data_Sheet_1.docx]

Supplementary Material

Appendix A

| *How well are the following services responding to the mental health care needs of the elderly (N=65)* | *Not*  *at all*  *N (%)* | *Low degree*  *N (%)* | *Some degree*  *N (%)* | *High degree*  *N (%)* | *Very high degree*  *N (%)* |
| --- | --- | --- | --- | --- | --- |
| *Access to specialized services* |  |  |  |  |  |
| difficulty accessing specialized resources | 5 (7.7) | 5 (7.7) | 17 (26.2) | 19 (29.2) | 19 (29.2) |
| scarcity of mental healthcare workers | 5 (7.7) | 5 (7.7) | 18 (27.7) | 19 (29.2 | 18 (27.7) |
| inadequate support and communication among stakeholders | - | 5 (7.7) | 18 (27.7 | 18 (27.7) | 24 (36.9) |
| lack of critical mental healthcare resources | 2 (3.1) | 4 (6.2) | 17 (26.2) | 23 (35.4) | 19 (29.2) |
| *Communication with mental health care providers* |  |  |  |  |  |
| direct communication with psychiatrists | 1 (1.5) | 8 (12.3) | 18 (27.7) | 22 (33.8) | 16 (24.6) |
| direct communication with psychologists | 2 (3.1) | 6 (9.2) | 14 (21.5) | 19 (29.2) | 24 (36.9) |
| long waiting times to see psychiatrists | 2 (3.1) | 9 (13.8) | 17 (26.2) | 17 (26.2) | 20 (30.8) |
| the referral process is too inefficient, burdensome and (or bureaucratic | 5 (7.7) | 1 (1.5) | 16 (24.6) | 24 (36.9) | 19 (29.2) |
| lack of explicitness in the roles of different healthcare professionals (e.g. GPs, psychiatrists, psychologists) in managing mental conditions | - | 5 (7.7) | 14 (21.5) | 20 (30.8) | 26 (40.0) |
| lack of support from mental healthcare teams | 1 (1.5) | 3 (4.6) | 14 (21.5) | 19 (29.2) | 28 (43.1) |
| *Personal and professional resources* |  |  |  |  |  |
| it requires a lot of time ( appointments had to be added to GPs' heavy schedules | 4 (6.2) | 14 (21.5) | 19 (29.2) | 12 (18.5) | 16 (24.6) |
| it requires flexibility | 6 (9.2) | 8 (12.3) | 22 (33.8) | 14 (21.5) | 15 (23.1) |
| it requires emotional investment | 3 (4.6) | 9 (13.8) | 20 (30.8) | 18 (27.7) | 15 (23.1) |
| *Patient related factors* |  |  |  |  |  |
| cases are too complex | 3 (4.6) | 7 (10.8) | 19 (29.2) | 21 (32.3) | 15 (23.1) |
| patient management is more taxing (e.g. poorer compliance to treatment and appointments, a disturbing presence in the waiting room) | 3 (4.6) | 12 (18.5) | 22 (33.8) | 11 (16.9) | 17 (26.2) |
| *Financial related factors* |  |  |  |  |  |
| inadequate level of remuneration | 6 (9.2) | 6 (9.2) | 13 (20.0) | 14 (21.5) | 26 (40.0) |
| increased bureaucracy to get reimbursed | 3 (4.6) | 6 (9.2) | 14 (21.5) | 15 (23.1) | 27 (41.5) |
| investing in education, time and effort to achieve knowledge and experience | 4 (6.2) | 8 (12.3) | 15 (23.1) | 13 (20.0) | 25 (38.5) |
| lack of financial motivation. | 3 (4.6) | 8 (12.3) | 19 (29.2) | 19 (29.2) | 16 (24.6) |
| *Competencies* |  |  |  |  |  |
| lack of available specialization programs | 2 (3.1) | 4 (6.2) | 16 (24.6) | 25 (38.5) | 18 (27.7) |
| elderly patients' common mental disorders (e.g. depression, anxiety) are beyond my ability to treat | 18 (27.7) | 14 (21.5) | 19 (29.2) | 6 (9.2) | 8 (12.3) |
| elderly patients' severe mental disorders (schizophrenia) are beyond my ability to treat | 1 (1.5) | 8 (12.3) | 17 (26.2) | 14 (21.5) | 25 (38.5) |
| elderly patients' neurocognitive problems (e.g. dementia) are beyond my ability to treat | 5 (7.7) | 10 (15.4) | 21 (32.3) | 15 (23.1) | 14 (21.5) |
| concern about the legal responsibility | 1 (1.5) | 5 (7.7) | 22 (33.8) | 15 (23.1) | 22 (33.8) |
| limited possibility to prescribe medication | 2 (3.1) | 2 (3.1) | 7 (10.8) | 20 (30.8) | 34 (52.3) |
| lack of self-confidence in diagnosing and managing mental conditions | 9 (13.8) | 17 (26.2) | 22 (33.8) | 11 (16.9) | 6 (9.2) |
| lack of guidelines of diagnosis and treatment of mental conditions | 4 (6.2) | 14 (21.5) | 13 (20.0) | 12 (18.5) | 22 (33.8) |
| uncertainty of the diagnosis | 1 (1.5) | 7 (10.8) | 14 (21.5) | 14 (21.5) | 29 (44.6) |
| uncertainty of the strategy to manage the patients’ conditions | 1 (1.5) | 10 (15.4) | 16 (24.6) | 19 (29.2) | 19 (29.2) |
| deterioration of the status of the patient | 1 (1.5) | 13 (20.0) | 17.26.2) | 16 (24.6) | 18 (27.7) |

Appendix B

| *Areas of care where GPs would be willing to get involved* | *Not at all or*  *to a low degree*  *N(%)* | *Some degree*  *N(%)* | *High or very high degree*  *N(%)* |
| --- | --- | --- | --- |
| **Early recognition and indicated prevention** |  |  |  |
| use of a screening instrument | 2 (3.1) | 11 (16.9) | 52 (80.0) |
| **Self-management and e-health interventions** |  |  |  |
| offer support for self-management | 5 (7.7) | 13 (20.0) | 47 (72.3) |
| prescribe e-health interventions | 17 (26.2) | 18 (27.7) | 30 (46.2) |
| **Diagnosis and treatment** |  |  |  |
| *Diagnosis and symptom severity* |  |  |  |
| assess the severity of the symptoms before treatment | 10 (15.4) | 14 (21.5) | 41 (63.1) |
| *Applying basic interventions* |  |  |  |
| provide educational material for patients | 4 (6.2) | 7 (10.8) | 54 (83.1) |
| *Providing stepped care treatment* |  |  |  |
| provide brief medical interventions | 15 (23.1) | 8 (12.3) | 42 (64.6) |
| provide psychological counselling | 25 (38.5) | 6 (9.2) | 34 (52.3) |
| provide psychiatric treatment – currently allowed | 6 (9.2) | 7 (10.8) | 52 (80.0) |
| provide psychiatric treatment – based on future certification | 12 (18.5) | 13 (20.0) | 40 (61.5) |
| systematically monitoring changes in the severity of symptoms with a validated instrument | 3 (4.6) | 16 (24.6) | 46 (70.8) |
| **Disease management and collaborative care** |  |  |  |
| make collaborative care agreements when other health care providers | 7 (10.8) | 10 (15.4) | 48 (73.8) |
| make agreements about referral of patients from secondary mental health care to primary care | 8 (12.3) | 6 (9.2) | 51 (78.5) |
| **Relapse prevention, rehabilitation and participation** |  |  |  |
| provide relapse prevention | 7 (10.8) | 14 (21.5) | 44 (67.7) |
| provide ongoing counselling to patients returning from secondary care | 11 (16.9) | 13 (20.0) | 41 (63.1) |
